# Weight loss as a predictor of cancer and serious disease in primary care: an ISAC-approved CPRD protocol for a retrospective cohort study using routinely collected primary care data from the UK

**DOI:** 10.1186/s41512-017-0019-9

**Published:** 2018-01-10

**Authors:** B. D. Nicholson, P. Aveyard, F. D. R. Hobbs, M. Smith, A. Fuller, R. Perera, W. Hamilton, S. Stevens, C. R. Bankhead

**Affiliations:** 1 Nuffield Department of Primary Care Health Sciences, Radcliffe Observatory Quarter, University of Oxford, Oxford, OX2 6GG UK; 2 University of Exeter, Medical School, St Luke’s Campus, Exeter, EX1 2LU UK

**Keywords:** Weight loss, Early detection of cancer, Serious disease, Primary care, Cohort study

## Abstract

**Background:**

Unexpected weight loss is a symptom of serious disease in primary care, for example between 1 in 200 and 1 in 30 patients with unexpected weight loss go on to develop cancer. However, it remains unclear how and when general practitioners (GPs) should investigate unexpected weight loss. Without clarification, GPs may wait too long before referring (choosing to watch and wait and potentially missing a diagnosis) or not long enough (overburdening hospital services and exposing patients to the risks of investigation). The overall aim of this study is to provide the evidence necessary to allow GPs to more effectively manage patients with unexpected weight loss.

**Methods:**

A retrospective cohort analysis of UK Clinical Practice Research Datalink (CPRD) data to: (1) describe how often in UK primary care the symptom of reported weight loss is coded, when weight is measured, and how GPs respond to a patient attending with unexpected weight loss; (2) identify the predictive value of recorded weight loss for cancer and serious disease in primary care, using cumulative incidence plots to compare outcomes between subgroups and Cox regression to explore and adjust for covariates. Preliminary work in CPRD estimates that weight loss as a symptom is recorded for approximately 148,000 eligible patients > 18 years and is distributed evenly across decades of age, providing adequate statistical power and precision in relation to cancer overall and common cancers individually. Further stratification by cancer stage will be attempted but may not be possible as not all practices within CPRD are eligible for cancer registry linkage, and staging information is often incomplete. The feasibility of using multiple imputation to address missing covariate values will be explored.

**Discussion:**

This will be the largest reported retrospective cohort of primary care patients with weight measurements and unexpected weight loss codes used to understand the association between weight measurement, unexpected weight loss, and serious disease including cancer. Our findings will directly inform international guidelines for the management of unexpected weight loss in primary care populations.

## Background

A 2014 systematic review suggests that the positive predictive value (PPV) for cancer is 33% in patients with an unexpected 10% loss of weight from baseline over 6–12 months. The same review reported a wide range of differential diagnoses for patients with unexpected weight loss, including advanced heart failure, chronic obstructive pulmonary disease, renal disease, pancreatic insufficiency, malabsorption, and endocrine disease, with up to 25% of patients without a diagnosis to explain their weight loss after extended follow-up [1]. However, these data mainly come from hospital inpatient populations or patients referred to the outpatient clinic where the prevalence of cancer and serious disease is much higher than in primary care as GPs have already filtered out many cases of weight loss that are more likely to be attributable to another cause. Given the absence of appropriate clinical guidelines or standardised practice, clinicians have been reported to take a wide range of action in response to patients with unexpected weight loss, from doing nothing through to ordering “extensive blind investigations” because of the fear of underlying cancer [2].

On the basis of primary care research, NICE (2015) has since suggested that unexpected weight loss is a sign of seven cancers, citing evidence from 14 studies reporting positive predictive values (PPVs) of 0.4–3% [3]. The problem for GPs is how to interpret and implement the term weight loss in these cancer guidelines: NICE do not define the degree of weight loss, or the time period of loss, that should prompt referral. Most cited studies referred to in the NICE guidelines define weight loss on the basis of a coded entry in the GP record, often based on a report of weight loss (volunteered by, or elicited from, the patient) rather than measured weight change [4–6]. Only one study referred to by NICE quantified the degree of weight loss that predicts colorectal cancer in primary care reporting odds ratios of 1.2 (95% CI 0.99–1.5) for 5–9.9% and 2.5 (2.1–3) for ≥ 10% weight loss [7]. However, in this study, weight loss was defined by comparing the last recorded weight with the highest recorded weight in the preceding 2 years [7], as weight is not routinely recorded in primary care and is considered a common missing variable in primary care databases [8].

There is an evidence gap for a comprehensive study to describe the use of weight measurement and coding for unexpected weight loss in primary care and for a study that determines the association between unexpected weight loss and cancer and serious disease that may lead to a comprehensive recommendation for the investigation of unexpected weight loss in primary care.

## Objective

The overall objective is to provide the evidence necessary to allow GPs to more effectively manage unexpected weight loss.

### Aims and rationale

#### Aim 1.1

To describe how often and when weight is measured, and the symptom of unexpected weight loss recorded as a code, in adults aged > 18 years, in NHS primary care.

#### Aim 1.2

To describe what action is taken in response to unexpected weight loss, in adults aged > 18 years, in NHS primary care.

Weight measurements and weight loss codes will be categorised using a rule-based search strategy developed as part of this project to identify the clinical purpose and clinical condition related to each weight entry in the primary care record, and the investigations requested, medications prescribed, and referrals made in response to the symptom of weight loss.

#### Aim 2.1

To identify the predictive value of unexpected weight loss recorded as a symptom for cancer in primary care in adults aged > 18 years.

#### Aim 2.2

If the symptom of unexpected weight loss predicts cancer, to explore if it is (i) independent of other symptoms, signs, and test results and (ii) restricted to late-stage disease.

#### Aim 2.3

To ascertain the predictive value of unexpected weight loss recorded as a symptom for serious disease in primary care.

The evidence regarding the predictive value of unexpected weight loss for cancer in primary care, which underpins the 2015 NICE guideline, does not cover all cancer types or take cancer stage at diagnosis into account. We will identify the predictive value of unexpected weight loss in primary care across all cancer types, explore the incremental predictive value of symptom combinations, and examine the association with cancer stage at diagnosis using a matched open cohort study design. In cases where cancer is excluded, an understanding of which alternative diagnoses are related to unexpected weight loss will inform subsequent management decisions in primary care. We will therefore identify the disease groups for which unexpected weight loss is also predictive to develop clinical guidance for the investigation of unexpected weight loss in primary care.

## Study type

### Aim 1: Descriptive

The descriptive epidemiology of weight measurement and weight loss coding in NHS primary care.

### Aims 2.1 and 2.3: Hypothesis testing

A cohort study of weight loss as a sign of cancer and serious disease in NHS primary care.

### Aim 2.2: Exploratory

Exploratory analysis to investigate the influence of covariates on the relationship between weight loss and the occurrence of cancer and serious disease.

## Study design

### The design of the study is an open cohort study.

## Sample size

In preparing this ISAC application, a preliminary search of 20 GP practices from 2000 to 2013 was conducted. Of 127,024 patients > 40 years with acceptable records, 80,562 (63.4%) had at least one weight measurement recorded during that period, 30,728 (24.1%) had two weight measurements within 6 months of each other, and 40,436 (31.8%) within 1 year; 3079 (2.4%) of patients had a Read code for weight loss but only half of these had an accompanying weight measurement.

Two thousand one hundred eighty-four patients with weight loss are required to detect a hazard ratio of 2 (a change in incidence of 1.5 to 3%) at 99% power (0.05% alpha) using a ratio of one case to five controls. It is anticipated that the study will therefore have sufficient power for stratification by cancer type, cancer stage, and using symptom combinations even though linkage to cancer registry may only be possible in approximately 60% of cancer cases [9].

Preliminary work in Clinical Practice Research Datalink (CPRD) estimated that that unexpected weight loss is coded as a symptom for about approximately 148,000 patients > 18 years and is distributed evenly across decades of age providing adequate statistical power and precision for a comprehensive cohort study investigating cancer and serious disease in adults (> 18 years). For example, if 3% of patients with weight loss develop cancer the number of Events Per Variable will far exceed the minimum number required for robust statistical modelling.

## Data linkage

### NCDR Cancer Registry Data

Linkage to the cancer register is required as cancer is a major outcome variable in this cohort study. Cancer registry data will provide more accurate information on cancer site and stage than reliance on the primary care record.

### Office of National Statistics (ONS) mortality data

Linkage is required to cross-validate cause of death for patients confirmed to have died of cancer using Cancer Registry Data linkage and to identify or confirm the cause of death in patients with and without serious disease as identified by the GP record.

### Index of Multiple Deprivation (IMD) scores

They are required to provide a GP (and where possible patient) level proxy for socioeconomic status to be used when describing both the baseline characteristics in the descriptive analysis of Aim 1 and the cohort analysis of Aim 2. IMD score will also be used as a covariate in the multivariate cox regression analysis as part of Aim 2 (see below).

## Study population

**Fig. 1:**
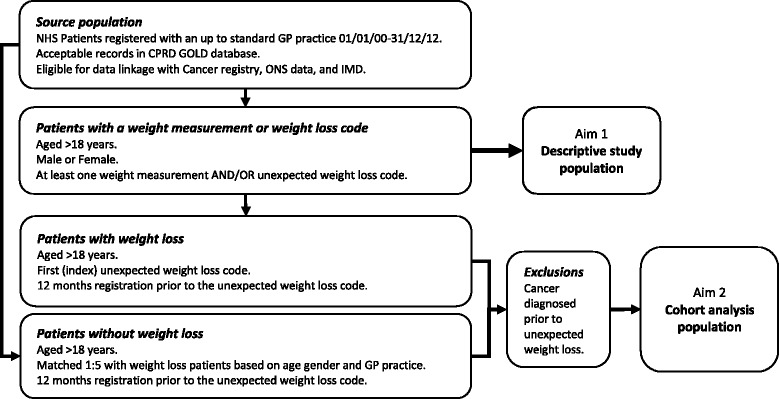
Flowchart of study populations

The study population is summarised in Fig. 1.

### Aim 1: Descriptive study

- NHS patients > 18 years
- Registered with a GP practice 1 January 2000–31 December 2011
- Eligible for data linkage with Cancer registry and ONS data

### Aim 2: Cohort analysis

Inclusions:

- NHS patients > 18 years
- Registered with a GP practice 1 January 2000–31 December 2011
- Eligible for data linkage with Cancer registry and ONS data.
- Patients with one of the unexpected weight loss codes (defined in Table 1)

**Table 1:** Weight measurement and unexpected weight loss codes

Unexpected weight loss codes		
Medcode	Readcode	Readterm
126	22A6.00	O/E—underweight
654	1623	Weight decreasing
1581	162..00	Weight symptom
3647	R032.00	[D]Abnormal loss of weight
4663	1625	Abnormal weight loss
5812	1625.11	Abnormal weight loss—symptom
12,398	1D1A.00	Complaining of weight loss
12,530	R034800	[D]Underweight
14,764	162Z.00	Weight symptom NOS
22,005	2224	O/E—cachexic
24,068	R2y4.00	[D]Cachexia
32,914	22K3.00	Body Mass Index low K/M2
37,937	22A8.00	Weight loss from baseline weight
42,309	22A7.00	Baseline weight
53,801	R2y4z00	[D]Cachexia NOS
102,563	1627	Unintentional weight loss
Weight measurement codes		
2	22A..00	O/E—weight
8105	22K..00	Body mass index
9015	22K4.00	Body mass index 25–29—overweight
13,278	22K5.00	Body mass index 30+—obesity
21,520	22AZ.00	O/E—weight NOS
22,556	22K7.00	Body mass index 40+—severely obese
24,496	22K6.00	Body mass index less than 20
28,937	22K2.00	Body mass index high K/M2
28,946	22K1.00	Body mass index normal K/M2
44,291	22K8.00	Body mass index 20–24—normal
101,047	22K9.00	Body mass index centile
105,791	22K9000	Baseline body mass index centile
105,800	22KB.00	Baseline body mass index
107,231	22KA.00	Target body mass index

Exclusions:

- Patients with a diagnosis of cancer prior to the index symptom of weight loss.

## Selection of comparison group(s) or controls

### Aim 1: Descriptive study

-No comparison group is required.

### Aim 2: Cohort analysis

- A matched cohort of patients without weight loss—patients without a coded entry for weight loss will be matched for age and sex and selected from the population of patients registered with the same practice having consulted within ± 3 months of the index weight loss code.
- Matching for age and sex will ensure there are sufficient patients without weight loss in each age and sex strata.
- A 1:5 sampling ratio achieves the best balance between data cost and statistical power (see sample size).

## Exposures, outcomes and covariates

### Aim 1: Descriptive study

Outcome 1: Objective weight measurement—quantitative weight measurements.

Outcome 2: Weight loss code—Read Codes defined in Table 1.

Patients with objective weight measurements or the symptom of unexpected weight loss recorded using the following Medcodes and Read codes listed in Table 1.

### Aim 2: Cohort analysis

#### Exposure—weight loss

Patients with the symptom of weight loss recorded using the unexpected weight loss Medcodes and Read Codes listed in Table 1. Weight loss codes will be independently categorised for clinical relavence by four co-investigators based on the results of the descriptive analysis, then consensus reached through discussion.   

#### Outcome—cancer

A library of over 1600 Read Codes and ICD-10 codes (grouped by site—see Table 2) developed by Hamilton and colleagues will be reviewed, updated using Read Code searches, and validated through consensus amongst co-investigators. All new cancer diagnoses in the 24 months following the weight loss code will be identified in CPRD and linked cancer registry data. To inform this analysis, data will also be extracted on cancer stage, grade, tumour size, and histology at diagnosis.

**Table 2:** Cancer codes

Cancer	Read code	Description	Medcode	ICD 10
Bladder	B490.00	Malignant neoplasm of trigone of urinary bladder	38,862	C670
	B491.00	Malignant neoplasm of dome of urinary bladder	44,996	C671
	B492.00	Malignant neoplasm of lateral wall of urinary bladder	35,963	C672
	B493.00	Malignant neoplasm of anterior wall of urinary bladder	19,162	C673
	B494.00	Malignant neoplasm of posterior wall of urinary bladder	42,012	C674
	B495.00	Malignant neoplasm of bladder neck	41,571	C675
	B496.00	Malignant neoplasm of ureteric orifice	28,241	C676
	B497.00	Malignant neoplasm of urachus	42,023	C677
	B49y000	Malignant neoplasm, overlapping lesion of bladder	47,801	C678
	B49y.00	Malignant neoplasm of other site of urinary bladder	36,949	C679
	B49z.00	Malignant neoplasm of urinary bladder NOS	31,102	C679
Breast	B335200	Malignant neoplasm of skin of breast	30,543	C445
	B34..11	CA female breast	348	C50
	B34..00	Malignant neoplasm of female breast	3968	C50
	B340000	Malignant neoplasm of nipple of female breast	23,380	C500
	B340.00	Malignant neoplasm of nipple and areola of female breast	26,853	C500
	B340z00	Malignant neoplasm of nipple or areola of female breast nos	59,831	C500
	B340100	Malignant neoplasm of areola of female breast	64,686	C500
	B341.00	Malignant neoplasm of central part of female breast	31,546	C501
	B342.00	Malignant neoplasm of upper-inner quadrant of female breast	29,826	C502
	B343.00	Malignant neoplasm of lower-inner quadrant of female breast	45,222	C503
	B344.00	Malignant neoplasm of upper-outer quadrant of female breast	23,399	C504
	B345.00	Malignant neoplasm of lower-outer quadrant of female breast	42,070	C505
	B346.00	Malignant neoplasm of axillary tail of female breast	20,685	C506
	B34y000	Malignant neoplasm of ectopic site of female breast	95,057	C508
	B34yz00	Malignant neoplasm of other site of female breast nos	38,475	C509
	B34y.00	Malignant neoplasm of other site of female breast	56,715	C509
	B34z.00	Malignant neoplasm of female breast nos	9470	C509
Cervix	B410z00	Malignant neoplasm of endocervix nos	50,285	C530
	B410.00	Malignant neoplasm of endocervix	48,820	C530
	B410000	Malignant neoplasm of endocervical canal	57,235	C530
	B410100	Malignant neoplasm of endocervical gland	53,103	C530
	B411.00	Malignant neoplasm of exocervix	50,297	C531
	B412.00	Malignant neoplasm, overlapping lesion of cervix uteri	58,094	C538
	B41y100	Malignant neoplasm of squamocolumnar junction of cervix	57,719	C538
	B41y000	Malignant neoplasm of cervical stump	95,505	C538
	B41yz00	Malignant neoplasm of other site of cervix nos	43,435	C539
	B41z.00	Malignant neoplasm of cervix uteri nos	28,311	C539
	B41y.00	Malignant neoplasm of other site of cervix	32,955	C539
Colorectal	B134.11	Carcinoma of caecum	22,163	C180
	B134.00	Malignant neoplasm of caecum	3811	C180
	B136.00	Malignant neoplasm of ascending colon	10,946	C182
	B130.00	Malignant neoplasm of hepatic flexure of colon	9088	C183
	B131.00	Malignant neoplasm of transverse colon	6935	C184
	B137.00	Malignant neoplasm of splenic flexure of colon	18,619	C185
	B132.00	Malignant neoplasm of descending colon	10,864	C186
	B133.00	Malignant neoplasm of sigmoid colon	2815	C187
	B138.00	Malignant neoplasm, overlapping lesion of colon	93,478	C188
	B13y.00	Malignant neoplasm of other specified sites of colon	48,231	C189
	B13z.11	Colonic cancer	9118	C189
	B13z.00	Malignant neoplasm of colon nos	28,163	C189
	B140.00	Malignant neoplasm of rectosigmoid junction	27,855	C19
	B141.12	Rectal carcinoma	5901	C20
	B141.11	Carcinoma of rectum	7219	C20
	B141.00	Malignant neoplasm of rectum	1800	C20
	B14y.00	Malig neop other site rectum, rectosigmoid junction and anus	55,659	C218
	B14z.00	Malignant neoplasm rectum,rectosigmoid junction and anus nos	50,974	C218
	B1z0.11	Cancer of bowel	11,628	C260
	B18y200	Malignant neoplasm of mesorectum	30,165	C481
Larynx	B214.00	Malignant neoplasm, overlapping lesion of larynx	50,579	C328
	B21z.00	Malignant neoplasm of larynx NOS	9237	C329
	B21y.00	Malignant neoplasm of larynx, other specified site	26,813	C329
	B210.00	Malignant neoplasm of glottis	318	C320
	B215.00	Malignant neoplasm of epiglottis NOS	55,374	C321
	B211.00	Malignant neoplasm of supraglottis	26,165	C321
	B212.00	Malignant neoplasm of subglottis	22,441	C322
	B213z00	Malignant neoplasm of laryngeal cartilage NOS	97,332	C323
	B213000	Malignant neoplasm of arytenoid cartilage	63,460	C323
	B213.00	Malignant neoplasm of laryngeal cartilage	43,111	C323
	B213100	Malignant neoplasm of cricoid cartilage	37,805	C323
Thyroid	B213300	Malignant neoplasm of thyroid cartilage	47,862	C323
	B53..00	Malignant neoplasm of thyroid gland	5637	C73
Sarcoma	B150200	Primary angiosarcoma of liver	68,410	C223
	B1z1100	Fibrosarcoma of spleen	72,224	C261
	B30z000	Osteosarcoma	19,437	C419
	B339.00	Dermatofibrosarcoma protuberans	24,375	C449
	B33z000	Kaposi’s sarcoma of skin	27,931	C460
	B05z000	Kaposi’s sarcoma of palate	37,549	C462
	B6z0.00	Kaposi’s sarcoma of lymph nodes	50,290	C463
	B592X00	Kaposi’s sarcoma of multiple organs	65,466	C468
	Byu5300	[X]kaposi’s sarcoma, unspecified	93,665	C469
	B59zX00	Kaposi’s sarcoma, unspecified	49,525	C469
	B600000	Reticulosarcoma of unspecified site	60,242	C833
	B600100	Reticulosarcoma of lymph nodes of head, face, and neck	71,031	C833
	B600700	Reticulosarcoma of spleen	95,058	C833
	B600300	Reticulosarcoma of intra-abdominal lymph nodes	70,374	C833
	B600.00	Reticulosarcoma	1481	C839
	B601000	Lymphosarcoma of unspecified site	71,625	C850
	B601200	Lymphosarcoma of intrathoracic lymph nodes	62,380	C850
	B601.00	Lymphosarcoma	27,416	C850
	B601100	Lymphosarcoma of lymph nodes of head, face and neck	71,238	C850
	B601z00	Lymphosarcoma nos	63,723	C850
	B601300	Lymphosarcoma of intra-abdominal lymph nodes	64,670	C850
	B653.00	Myeloid sarcoma	70,724	C923
	B653100	Granulocytic sarcoma	39,629	C923
	B67y000	Lymphosarcoma cell leukaemia	72,197	C947
	B304200	Malignant neoplasm of humerus	61,741	C400
	B304000	Malignant neoplasm of scapula	49,054	C400
	B304300	Malignant neoplasm of radius	92,371	C400
	B304.00	Malignant neoplasm of scapula and long bones of upper arm	71,810	C400
	B304z00	Malignant neoplasm of scapula and long bones of upper arm NOS	65,880	C400
	B304400	Malignant neoplasm of ulna	64,848	C400
	B305.00	Malignant neoplasm of hand bones	73,530	C401
	B305.12	Malignant neoplasm of metacarpal bones	72,464	C401
	B305C00	Malignant neoplasm of fifth metacarpal bone	94,427	C401
	B305z00	Malignant neoplasm of hand bones NOS	73,556	C401
	B305100	Malignant neoplasm of carpal bone—lunate	69,104	C401
	B305000	Malignant neoplasm of carpal bone—scaphoid	57,988	C401
	B305D00	Malignant neoplasm of phalanges of hand	86,812	C401
	B307z00	Malignant neoplasm of long bones of leg NOS	62,630	C402
	B307.00	Malignant neoplasm of long bones of leg	68,055	C402
	B307200	Malignant neoplasm of tibia	40,814	C402
	B307100	Malignant neoplasm of fibula	50,402	C402
	B307000	Malignant neoplasm of femur	56,513	C402
	B308300	Malignant neoplasm of medial cuneiform	34,878	C403
	B308800	Malignant neoplasm of first metatarsal bone	69,927	C403
	B308B00	Malignant neoplasm of fourth metatarsal bone	92,382	C403
	B308100	Malignant neoplasm of talus	95,182	C403
	B308D00	Malignant neoplasm of phalanges of foot	58,949	C403
	B308200	Malignant neoplasm of calcaneum	72,212	C403
	B30X.00	Malignant neoplasm/bones + articular cartilage/limb, unspecified	43,614	C409
	Byu3100	[X]Malignant neoplasm/bones + articular cartilage/limb, unspecified	73,296	C409
	B300600	Malignant neoplasm of parietal bone	54,747	C410
	B300400	Malignant neoplasm of occipital bone	55,953	C410
	B300z00	Malignant neoplasm of bones of skull and face NOS	69,146	C410
	B300300	Malignant neoplasm of nasal bone	95,458	C410
	B300900	Malignant neoplasm of zygomatic bone	50,299	C410
	B300C00	Malignant neoplasm of vomer	44,452	C410
	B300500	Malignant neoplasm of orbital bone	50,298	C410
	B300700	Malignant neoplasm of sphenoid bone	55,595	C410
	B300200	Malignant neoplasm of malar bone	59,520	C410
	B300B00	Malignant neoplasm of turbinate	96,445	C410
	B300000	Malignant neoplasm of ethmoid bone	53,594	C410
	B300100	Malignant neoplasm of frontal bone	53,599	C410
	B300800	Malignant neoplasm of temporal bone	62,104	C410
	B300.00	Malignant neoplasm of bones of skull and face	59,036	C410
	B300A00	Malignant neoplasm of maxilla	17,475	C410
	B301.00	Malignant neoplasm of mandible	33,833	C411
	B302100	Malignant neoplasm of thoracic vertebra	32,372	C412
	B302.00	Malignant neoplasm of vertebral column	16,704	C412
	B302000	Malignant neoplasm of cervical vertebra	46,939	C412
	B302200	Malignant neoplasm of lumbar vertebra	54,691	C412
	B302z00	Malignant neoplasm of vertebral column NOS	49,701	C412
	B303000	Malignant neoplasm of rib	37,842	C413
	B303.00	Malignant neoplasm of ribs, sternum and clavicle	27,528	C413
	B303100	Malignant neoplasm of sternum	49,491	C413
	B303z00	Malignant neoplasm of rib, sternum and clavicle NOS	51,237	C413
	B303500	Malignant neoplasm of xiphoid process	54,493	C413
	B303300	Malignant neoplasm of costal cartilage	60,403	C413
	B303200	Malignant neoplasm of clavicle	66,639	C413
	B306.00	Malignant neoplasm of pelvic bones, sacrum and coccyx	54,631	C414
	B306100	Malignant neoplasm of ischium	59,223	C414
	B306400	Malignant neoplasm of coccygeal vertebra	66,908	C414
	B306z00	Malignant neoplasm of pelvis, sacrum or coccyx NOS	38,938	C414
	B306300	Malignant neoplasm of sacral vertebra	40,966	C414
	B306200	Malignant neoplasm of pubis	51,921	C414
	B306000	Malignant neoplasm of ilium	44,609	C414
	Byu3200	[X]Malignant neoplasm/overlap lesion/bone + articular cartilage	63,300	C418
	B30W.00	Malignant neoplasm/overlap lesion/bone + articular cartilage	67,451	C418
	B303400	Malignant neoplasm of costo-vertebral joint	67,763	C418
	B30z.00	Malignant neoplasm of bone and articular cartilage NOS	16,075	C419
	Byu3300	[X]Malignant neoplasm/bone + articular cartilage, unspecified	43,151	C419
	B310z00	Malig neop connective and soft tissue head, face, neck NOS	73,718	C490
	B310100	Malignant neoplasm of soft tissue of face	40,014	C490
	B310000	Malignant neoplasm of soft tissue of head	59,382	C490
	B310300	Malignant neoplasm of cartilage of ear	60,035	C490
	B310.00	Malignant neoplasm of connective and soft tissue head, face and neck	43,475	C490
	B310200	Malignant neoplasm of soft tissue of neck	48,517	C490
	B310400	Malignant neoplasm of tarsus of eyelid	49,463	C490
	B311500	Malignant neoplasm of connective and soft tissue of thumb	63,988	C491
	B311200	Malignant neoplasm of connective and soft tissue of fore-arm	57,482	C491
	B311100	Malignant neoplasm of connective and soft tissue, upper arm	64,345	C491
	B311000	Malignant neoplasm of connective and soft tissue of shoulder	50,222	C491
	B311400	Malignant neoplasm of connective and soft tissue of finger	91,586	C491
	B311300	Malignant neoplasm of connective and soft tissue of hand	19,321	C491
	B311.00	Malignant neoplasm connective and soft tissue upper limb/shoulder	53,989	C491
	B312300	Malignant neoplasm of connective and soft tissue of lower leg	30,542	C492
	B312400	Malignant neoplasm of connective and soft tissue of foot	54,222	C492
	B312.00	Malignant neoplasm of connective and soft tissue of hip and leg	66,088	C492
	B312z00	Malignant neoplasm connective and soft tissue hip and leg NOS	90,546	C492
	B312200	Malignant neoplasm connective and soft tissue of popliteal space	54,965	C492
	B312100	Malignant neoplasm of connective and soft tissue thigh and upper leg	44,805	C492
	B313100	Malignant neoplasm of diaphragm	54,186	C493
	B313.00	Malignant neoplasm of connective and soft tissue of thorax	22,290	C493
	B313000	Malignant neoplasm of connective and soft tissue of axilla	29,160	C493
	B313200	Malignant neoplasm of great vessels	72,522	C493
	B314.00	Malignant neoplasm of connective and soft tissue of abdomen	45,071	C494
	B314z00	Malignant neoplasm of connective and soft tissue of abdomen NOS	60,247	C494
	B314000	Malignant neoplasm of connective and soft tissue of abdominal wall	66,488	C494
	B315z00	Malignant neoplasm of connective and soft tissue of pelvis NOS	58,836	C495
	B315000	Malignant neoplasm of connective and soft tissue of buttock	70,463	C495
	B315200	Malignant neoplasm of connective and soft tissue of perineum	59,152	C495
	B315.00	Malignant neoplasm of connective and soft tissue of pelvis	51,965	C495
	B315100	Malignant neoplasm of connective and soft tissue of inguinal region	67,324	C495
	Byu5800	[X]Mal neoplasm/connective + soft tissue of trunk, unspecified	91,896	C496
	B314100	Malig neoplasm of connective and soft tissues of lumb spine	94,272	C496
	B316.00	Malig neop of connective and soft tissue trunk unspecified	57,471	C496
	B31z.00	Malignant neoplasm of connective and soft tissue, site NOS	15,182	C499
	Byu5900	[X]Malignant neoplasm/connective + soft tissue, unspecified	91,457	C499
	B31y.00	Malignant neoplasm connective and soft tissue other specified site	65,233	C499
Kidney	B4A0.00	Malignant neoplasm of kidney parenchyma	1599	C64
	B4A..11	Renal malignant neoplasm	18,712	C64
	B4A..00	Malignant neoplasm of kidney and other unspecified urinary organs	13,559	C64
	B4A0000	Hypernephroma	7978	C64
	B4A1000	Malignant neoplasm of renal calyces	27,540	C65
	B4A1z00	Malignant neoplasm of renal pelvis NOS	54,184	C65
	B4A1.00	Malignant neoplasm of renal pelvis	12,389	C65
	B4Az.00	Malignant neoplasm of kidney or urinary organs NOS	29,462	C689
Lung	B221100	Malignant neoplasm of hilus of lung	33,444	C340
	B221.00	Malignant neoplasm of main bronchus	12,870	C340
	B221z00	Malignant neoplasm of main bronchus NOS	21,698	C340
	B221000	Malignant neoplasm of carina of bronchus	17,391	C340
	B222.11	Pancoast’s syndrome	20,170	C341
	B222.00	Malignant neoplasm of upper lobe, bronchus or lung	10,358	C341
	B222000	Malignant neoplasm of upper lobe bronchus	31,700	C341
	B222100	Malignant neoplasm of upper lobe of lung	25,886	C341
	B222z00	Malignant neoplasm of upper lobe, bronchus or lung NOS	44,169	C341
	B223100	Malignant neoplasm of middle lobe of lung	39,923	C342
	B223z00	Malignant neoplasm of middle lobe, bronchus or lung NOS	54,134	C342
	B223.00	Malignant neoplasm of middle lobe, bronchus or lung	31,268	C342
	B223000	Malignant neoplasm of middle lobe bronchus	41,523	C342
	B224z00	Malignant neoplasm of lower lobe, bronchus or lung NOS	42,566	C343
	B224100	Malignant neoplasm of lower lobe of lung	12,582	C343
	B224000	Malignant neoplasm of lower lobe bronchus	18,678	C343
	B224.00	Malignant neoplasm of lower lobe, bronchus or lung	31,188	C343
	B225.00	Malignant neoplasm of overlapping lesion of bronchus and lung	36,371	C348
	B22z.00	Malignant neoplasm of bronchus or lung NOS	3903	C349
	Byu2000	[X]malignant neoplasm of bronchus or lung, unspecified	40,595	C349
	B22z.11	Lung cancer	2587	C349
	B22y.00	Malignant neoplasm of other sites of bronchus or lung	38,961	C349
	B26..00	Malignant neoplasm, overlap lesion of resp and intrathor orgs	66,646	C398
	B2zy.00	Malignant neoplasm of other site of respiratory tract	29,283	C399
Hodgkins lymphoma	B613.00	Hodgkin’s disease, lymphocytic-histiocytic predominance	38,939	C810
	B613600	Hodgkin’s, lymphocytic-histiocytic pred intrapelvic nodes	95,338	C810
	B613z00	Hodgkin’s, lymphocytic-histiocytic predominance nos	29,876	C810
	B613300	Hodgkin’s, lymphocytic-histiocytic pred intra-abdominal node	73,532	C810
	B613000	Hodgkin’s, lymphocytic-histiocytic predominance unspec site	71,142	C810
	B613200	Hodgkin’s, lymphocytic-histiocytic pred intrathoracic nodes	92,245	C810
	B613100	Hodgkin’s, lymphocytic-histiocytic pred of head, face, neck	68,330	C810
	B613500	Hodgkin’s, lymphocytic-histiocytic pred inguinal and leg	93,951	C810
	B614400	Hodgkin’s nodular sclerosis of lymph nodes of axilla and arm	65,483	C811
	B614300	Hodgkin’s nodular sclerosis of intra-abdominal lymph nodes	61,149	C811
	B614.00	Hodgkin’s disease, nodular sclerosis	29,178	C811
	B614100	Hodgkin’s nodular sclerosis of head, face and neck	55,303	C811
	B614z00	Hodgkin’s disease, nodular sclerosis NOS	63,054	C811
	B614200	Hodgkin’s nodular sclerosis of intrathoracic lymph nodes	67,506	C811
	B614000	Hodgkin’s disease, nodular sclerosis of unspecified site	57,225	C811
	B614800	Hodgkin’s nodular sclerosis of lymph nodes of multiple sites	19,140	C811
	B615200	Hodgkin’s mixed cellularity of intrathoracic lymph nodes	58,684	C812
	B615z00	Hodgkin’s disease, mixed cellularity NOS	94,005	C812
	B615.00	Hodgkin’s disease, mixed cellularity	49,605	C812
	B615100	Hodgkin’s mixed cellularity of lymph nodes head, face, neck	94,407	C812
	B615000	Hodgkin’s disease, mixed cellularity of unspecified site	97,863	C812
	B616.00	Hodgkin’s disease, lymphocytic depletion	67,703	C813
	B616400	Hodgkin’s lymphocytic depletion lymph nodes axilla and arm	63,625	C813
	B616000	Hodgkin’s lymphocytic depletion of unspecified site	95,049	C813
	ByuD000	[X]other Hodgkin’s disease	43,415	C817
	B610.00	Hodgkin’s paragranuloma	65,489	C817
	B611.00	Hodgkin’s granuloma	44,196	C817
	B61z100	Hodgkin’s disease NOS of lymph nodes of head, face and neck	59,778	C819
	B61..00	Hodgkin’s disease	2462	C819
	B61zz00	Hodgkin’s disease NOS	42,461	C819
	B61z800	Hodgkin’s disease NOS of lymph nodes of multiple sites	97,746	C819
	B61z200	Hodgkin’s disease NOS of intrathoracic lymph nodes	59,755	C819
	B61z.00	Hodgkin’s disease NOS	53,397	C819
	B61z000	Hodgkin’s disease NOS, unspecified site	61,662	C819
	B61z400	Hodgkin’s disease NOS of lymph nodes of axilla and arm	91,900	C819
	B61z700	Hodgkin’s disease NOS of spleen	94,279	C819
	B612.00	Hodgkin’s sarcoma	64,036	C817
	B612400	Hodgkin’s sarcoma of lymph nodes of axilla and upper limb	68,039	C817
Non-Hodgkins lymphoma	B627000	Follicular non-Hodgkin’s small cleaved cell lymphoma	28,639	C820
	B627100	Follicular non-Hodgkin’s mixed sml cleavd & lge cell lymphoma	70,842	C821
	B627200	Follicular non-Hodgkin’s large cell lymphoma	49,262	C822
	B627B00	Other types of follicular non-Hodgkin’s lymphoma	31,576	C827
	ByuD100	[X]other types of follicular non-Hodgkin’s lymphoma	67,518	C827
	B620500	Nodular lymphoma of lymph nodes of inguinal region and leg	94,995	C829
	B627C11	Follicular lymphoma NOS	17,182	C829
	B620000	Nodular lymphoma of unspecified site	66,327	C829
	B620100	Nodular lymphoma of lymph nodes of head, face and neck	45,264	C829
	B620z00	Nodular lymphoma NOS	65,701	C829
	B620.00	Nodular lymphoma (brill - symmers disease)	5179	C829
	B620300	Nodular lymphoma of intra-abdominal lymph nodes	92,068	C829
	B627C00	Follicular non-Hodgkin’s lymphoma	21,549	C829
	B620800	Nodular lymphoma of lymph nodes of multiple sites	58,082	C829
	B627300	Diffuse non-Hodgkin’s small cell (diffuse) lymphoma	50,668	C830
	B627500	Diffuse non-Hodgkin mixed small & large cell (diffuse) lymphoma	50,695	C832
	B627600	Diffuse non-Hodgkin’s immunoblastic (diffuse) lymphoma	53,551	C834
	B627700	Diffuse non-Hodgkin’s lymphoblastic (diffuse) lymphoma	17,460	C835
	B627800	Diffuse non-Hodgkin’s lymphoma undifferentiated (diffuse)	65,180	C836
	B602300	Burkitt’s lymphoma of intra-abdominal lymph nodes	97,577	C837
	B602z00	Burkitt’s lymphoma NOS	71,304	C837
	B602.00	Burkitt’s lymphoma	21,402	C837
	B602500	Burkitt’s lymphoma of lymph nodes of inguinal region and leg	92,380	C837
	B602100	Burkitt’s lymphoma of lymph nodes of head, face and neck	59,115	C837
	B627D00	Diffuse non-Hodgkin’s centroblastic lymphoma	70,509	C838
	ByuDC00	[X]Diffuse non-Hodgkin’s lymphoma, unspecified	64,515	C839
	B627X00	Diffuse non-Hodgkin’s lymphoma, unspecified	39,798	C839
	B622.00	Sezary’s disease	35,014	C841
	B62x000	T-zone lymphoma	90,201	C842
	B62x100	Lymphoepithelioid lymphoma	57,737	C843
	B62x200	Peripheral t-cell lymphoma	12,464	C844
	B62xX00	Oth and unspecif peripheral and cutaneous t cell lymphomas	44,318	C845
	B627W00	Unspecified b-cell non-Hodgkin’s lymphoma	31,794	C851
	ByuDE00	[X]unspecified b-cell non-Hodgkin’s lymphoma	63,375	C851
	ByuD300	[X]Other specified types of non-Hodgkin’s lymphoma	64,336	C857
	B62y100	Malignant lymphoma NOS of lymph nodes of head, face and neck	50,696	C859
	B62y500	Malignant lymphoma NOS of lymph node inguinal region and leg	63,105	C859
	B62y400	Malignant lymphoma NOS of lymph nodes of axilla and arm	34,089	C859
	B62y000	Malignant lymphoma NOS of unspecified site	57,427	C859
	B62y700	Malignant lymphoma NOS of spleen	60,092	C859
	ByuDF11	[X]Non-Hodgkin’s lymphoma NOS	7940	C859
	B62y600	Malignant lymphoma NOS of intrapelvic lymph nodes	71,262	C859
	B62y200	Malignant lymphoma NOS of intrathoracic lymph nodes	72,725	C859
	B62yz00	Malignant lymphoma NOS	15,027	C859
	ByuDF00	[X]Non-Hodgkin’s lymphoma, unspecified type	8649	C859
	B62y.00	Malignant lymphoma NOS	12,335	C859
	B62y300	Malignant lymphoma NOS of intra-abdominal lymph nodes	42,579	C859
	B62x600	True histiocytic lymphoma	95,630	C963
	B6z..00	Malignant neoplasm lymphatic or haematopoietic tissue NOS	49,301	C969
	B62y800	Malignant lymphoma NOS of lymph nodes of multiple sites	15,504	C969
	B621000	Mycosis fungoides of unspecified site	95,949	C840
	B621500	Mycosis fungoides of lymph nodes of inguinal region and leg	72,714	C840
	B621.00	Mycosis fungoides	12,006	C840
	B621800	Mycosis fungoides of lymph nodes of multiple sites	95,012	C840
	B621400	Mycosis fungoides of lymph nodes of axilla and upper limb	96,379	C840
	B621300	Mycosis fungoides of intra-abdominal lymph nodes	91,674	C840
	B621z00	Mycosis fungoides NOS	38,005	C840
	B62x400	Malignant reticulosis	62,437	C857
Melanoma	B320.00	Malignant melanoma of lip	70,637	C430
	B321.00	Malignant melanoma of eyelid including canthus	54,632	C431
	B322000	Malignant melanoma of auricle (ear)	59,061	C432
	B322.00	Malignant melanoma of ear and external auricular canal	57,260	C432
	B322z00	Malignant melanoma of ear and external auricular canal NOS	73,744	C432
	B323100	Malignant melanoma of chin	71,136	C433
	B323200	Malignant melanoma of eyebrow	47,094	C433
	B323500	Malignant melanoma of temple	58,958	C433
	B323z00	Malignant melanoma of face NOS	67,806	C433
	Byu4000	[X]malignant melanoma of other + unspecified parts of face	56,925	C433
	B323.00	Malignant melanoma of other and unspecified parts of face	47,252	C433
	B323300	Malignant melanoma of forehead	68,133	C433
	B323400	Malignant melanoma of external surface of nose	45,139	C433
	B323000	Malignant melanoma of external surface of cheek	41,278	C433
	B324000	Malignant melanoma of scalp	55,881	C434
	B324.00	Malignant melanoma of scalp and neck	65,625	C434
	B324100	Malignant melanoma of neck	45,306	C434
	B325700	Malignant melanoma of back	43,463	C435
	B325800	Malignant melanoma of chest wall	51,209	C435
	B325600	Malignant melanoma of umbilicus	43,715	C435
	B325100	Malignant melanoma of breast	32,768	C435
	B325300	Malignant melanoma of groin	34,259	C435
	B325200	Malignant melanoma of buttock	53,629	C435
	B325500	Malignant melanoma of perineum	95,629	C435
	B325.00	Malignant melanoma of trunk (excluding scrotum)	38,689	C435
	B325z00	Malignant melanoma of trunk, excluding scrotum, NOS	45,760	C435
	B325000	Malignant melanoma of axilla	49,814	C435
	B326200	Malignant melanoma of fore-arm	45,755	C436
	B326400	Malignant melanoma of finger	25,602	C436
	B326300	Malignant melanoma of hand	62,475	C436
	B326000	Malignant melanoma of shoulder	50,505	C436
	B326500	Malignant melanoma of thumb	63,997	C436
	B326z00	Malignant melanoma of upper limb or shoulder NOS	55,292	C436
	B326100	Malignant melanoma of upper arm	54,685	C436
	B326.00	Malignant melanoma of upper limb and shoulder	65,164	C436
	B327500	Malignant melanoma of ankle	42,714	C437
	B327700	Malignant melanoma of foot	41,490	C437
	B327000	Malignant melanoma of hip	73,536	C437
	B327100	Malignant melanoma of thigh	51,873	C437
	B327800	Malignant melanoma of toe	36,899	C437
	B327200	Malignant melanoma of knee	54,305	C437
	B327.00	Malignant melanoma of lower limb and hip	46,255	C437
	B327600	Malignant melanoma of heel	61,246	C437
	B327300	Malignant melanoma of popliteal fossa area	39,878	C437
	B327z00	Malignant melanoma of lower limb or hip NOS	64,327	C437
	B327900	Malignant melanoma of great toe	53,369	C437
	B327400	Malignant melanoma of lower leg	37,872	C437
	B32y000	Overlapping malignant melanoma of skin	96,585	C438
	B32z.00	Malignant melanoma of skin NOS	28,556	C439
	Byu4100	[X]malignant melanoma of skin, unspecified	19,444	C439
	B32..00	Malignant melanoma of skin	865	C439
	B32y.00	Malignant melanoma of other specified skin site	42,153	C439
Myeloma	B63z.00	Immunoproliferative neoplasm or myeloma NOS	43,450	C889
	B630.12	Myelomatosis	15,211	C900
	B630.00	Multiple myeloma	4944	C900
	B630300	Lambda light chain myeloma	46,042	C900
	B631.00	Plasma cell leukaemia	39,187	C901
	B630100	Solitary myeloma	19,028	C902
	B630200	Plasmacytoma NOS	21,329	C902
	B630000	Malignant plasma cell neoplasm, extramedullary plasmacytoma	22,158	C902
Oesophagus	B100.00	Malignant neoplasm of cervical oesophagus	61,695	C150
	B101.00	Malignant neoplasm of thoracic oesophagus	41,362	C151
	B102.00	Malignant neoplasm of abdominal oesophagus	63,470	C152
	B103.00	Malignant neoplasm of upper third of oesophagus	50,789	C153
	B104.00	Malignant neoplasm of middle third of oesophagus	54,171	C154
	B105.00	Malignant neoplasm of lower third of oesophagus	42,416	C155
	B106.00	Malignant neoplasm, overlapping lesion of oesophagus	67,497	C158
	B10y.00	Malignant neoplasm of other specified part of oesophagus	53,591	C159
	B10z.00	Malignant neoplasm of oesophagus NOS	30,700	C159
	B10z.11	Oesophageal cancer	4865	C159
	B110111	Malignant neoplasm of gastro-oesophageal junction	94,278	C160
Ovary	B440.00	Malignant neoplasm of ovary	7805	C56
	B440.11	Cancer of ovary	1986	C56
	B44..00	Malignant neoplasm of ovary and other uterine adnexa	19,141	C578
Pancreas	B162.00	Malignant neoplasm of ampulla of vater	10,949	C241
	B170.00	Malignant neoplasm of head of pancreas	8771	C250
	B171.00	Malignant neoplasm of body of pancreas	40,810	C251
	B172.00	Malignant neoplasm of tail of pancreas	39,870	C252
	B173.00	Malignant neoplasm of pancreatic duct	35,535	C253
	B174.00	Malignant neoplasm of islets of langerhans	35,795	C254
	B17y.00	Malignant neoplasm of other specified sites of pancreas	48,537	C257
	B17yz00	Malignant neoplasm of specified site of pancreas NOS	95,783	C257
	B175.00	Malignant neoplasm, overlapping lesion of pancreas	97,875	C258
	B17y000	Malignant neoplasm of ectopic pancreatic tissue	96,635	C259
	B17z.00	Malignant neoplasm of pancreas NOS	34,388	C259
Prostate	B46..00	Malignant neoplasm of prostate	780	C61
Stomach	B110100	Malignant neoplasm of cardio-oesophageal junction of stomach	22,894	C160
	B110z00	Malignant neoplasm of cardia of stomach NOS	37,859	C160
	B110.00	Malignant neoplasm of cardia of stomach	32,022	C160
	B113.00	Malignant neoplasm of fundus of stomach	32,362	C161
	B114.00	Malignant neoplasm of body of stomach	43,572	C162
	B112.00	Malignant neoplasm of pyloric antrum of stomach	19,318	C163
	B111z00	Malignant neoplasm of pylorus of stomach NOS	59,092	C164
	B111100	Malignant neoplasm of pyloric canal of stomach	41,215	C164
	B111000	Malignant neoplasm of prepylorus of stomach	48,237	C164
	B111.00	Malignant neoplasm of pylorus of stomach	21,620	C164
	B115.00	Malignant neoplasm of lesser curve of stomach unspecified	42,193	C165
	B116.00	Malignant neoplasm of greater curve of stomach unspecified	55,434	C166
	B11y000	Malignant neoplasm of anterior wall of stomach nec	65,312	C168
	B11y100	Malignant neoplasm of posterior wall of stomach nec	96,802	C168
	B117.00	Malignant neoplasm, overlapping lesion of stomach	51,690	C168
	B11yz00	Malignant neoplasm of other specified site of stomach NOS	65,372	C169
	B11y.00	Malignant neoplasm of other specified site of stomach	55,019	C169
	B11z.00	Malignant neoplasm of stomach NOS	14,800	C169
Testis	B470200	Seminoma of undescended testis	7740	C620
	B470.00	Malignant neoplasm of undescended testis	64,602	C620
	B470300	Teratoma of undescended testis	36,325	C620
	B470z00	Malignant neoplasm of undescended testis NOS	96,429	C620
	B471z00	Malignant neoplasm of descended testis NOS	91,509	C621
	B471000	Seminoma of descended testis	21,786	C621
	B471100	Teratoma of descended testis	9476	C621
	B471.00	Malignant neoplasm of descended testis	19,475	C621
	B47z.00	Malignant neoplasm of testis NOS	38,510	C629
	B47z.11	Seminoma of testis	2961	C629
	B47z.12	Teratoma of testis	15,989	C629
	B48y100	Malignant neoplasm of tunica vaginalis	47,668	C637
Uterus	B431000	Malignant neoplasm of lower uterine segment	59,097	C540
	B431z00	Malignant neoplasm of isthmus of uterine body NOS	70,729	C540
	B431.00	Malignant neoplasm of isthmus of uterine body	43,940	C540
	B430211	Malignant neoplasm of endometrium	49,400	C541
	B430200	Malignant neoplasm of endometrium of corpus uteri	2890	C541
	B430300	Malignant neoplasm of myometrium of corpus uteri	45,793	C542
	B430100	Malignant neoplasm of fundus of corpus uteri	68,155	C543
	B432.00	Malignant neoplasm of overlapping lesion of corpus uteri	16,967	C548
	B43z.00	Malignant neoplasm of body of uterus NOS	33,617	C549
	B43y.00	Malignant neoplasm of other site of uterine body	31,608	C549
	B430000	Malignant neoplasm of cornu of corpus uteri	72,723	C549
	B430z00	Malignant neoplasm of corpus uteri NOS	45,490	C549
	B43..00	Malignant neoplasm of body of uterus	7046	C549
	B40..00	Malignant neoplasm of uterus, part unspecified	2744	C55
Vulval	B451.00	Malignant neoplasm of labia majora	43,761	C510
	B453.00	Malignant neoplasm of clitoris	53,910	C512
	B45y000	Malignant neoplasm of overlapping lesion of vulva	27,617	C518
	B454.00	Malignant neoplasm of vulva unspecified	4554	C519
	B454.11	Primary vulval cancer	11,991	C519
	B451z00	Malignant neoplasm of labia majora NOS	59,362	C510
	B451000	Malignant neoplasm of greater vestibular (Bartholin’s) gland	47,899	C510
	B452.00	Malignant neoplasm of labia minora	58,061	C511
Vaginal	B450.00	Malignant neoplasm of vagina	37,328	C52
	B450100	Malignant neoplasm of vaginal vault	10,698	C52
	B450z00	Malignant neoplasm of vagina NOS	60,772	C52

#### Outcome—serious disease

A library of candidate Read Codes for the most common serious diseases related to unexpected weight loss will be developed by combining two approaches: (i) review of the most frequent diagnostic codes entered in the clinical record within the period surrounding the unexpected weight loss code (descriptive study analysis section); (ii) review of the literature on causes of unexpected weight loss [1, 2]. A list of these candidate conditions will be reviewed independently by four co-investigators until consensus is reached on up to 20 serious diseases to be identified in the 24 months following the weight loss code.

#### Covariates

Data will also be extracted to explore the effect of the following factors which could independently impact the recording of weight and the occurrence of cancer:

1. Personal characteristics—age, gender, ethnicity, smoking history, alcohol intake, family history of cancer, and IMD score recorded before the date of the weight loss code (index date).
2. Co-morbidity—recorded before the index date (no time limit) or implied from the prescribing record at the index date.
3. Other cancer symptoms and signs—using Read Codes for symptoms shown to have an independent association with cancer as described by NICE [3]. These will be sought for 3 months before to 2 years after the index date.
4. Results of basic cancer investigations used routinely in primary care: CxR, FBC, LFTs (inc. alkaline phosphatase), calcium, PSA, CA125, and inflammatory markers. These will be sought for 3 months before to 2 years after the index date.

## Data/statistical analysis

### Aim 1: Descriptive study

To describe how often and when weight is recorded, we will request preliminary CPRD searches to identify all: (1) Read coded entries for weight loss and (2) quantitative weight measurements.

A subset of patients with weight measurements and unexpected weight loss codes will be used to develop a rule-based search strategy to categorise: (1) the clinical purpose (e.g. prevention, monitoring, diagnosis); (2) the related clinical condition (e.g. diabetes, heart failure, cancer). The GPs’ subsequent actions will be described in terms of (1) investigations requested, (2) medications prescribed, and (3) referrals made. The search strategy will then be applied to the entire cohort of weight measurements and weight loss codes.

The most effective method to identify the reason for the weight entry and the subsequent action will be investigated. For example, codelists will be developed to capture the clinical purpose of the consultation associated with each weight measurement or weight loss code: health check codes will be used to identify prevention activity; chronic disease review codes will be used to identify monitoring. For associated clinical conditions, symptom and diagnostic codes entered at the same time as each weight measurement or weight loss code will be ascertained and frequency ranked for the entire descriptive study population. Initially, searches will be performed on the day of the weight entry, then a sensitivity analysis will be performed increasing the time window to ± 1 day of the weight entry, then 1 week, 1 month, and so on. This strategy will be repeated to identify investigation and referral codes following entry of the weight loss code.

### Aim 2: Cohort analysis

#### Cumulative incidence plots

Cumulative incidence plots will be used to describe the probability of cancer or serious disease over time for those with and without weight loss. These will be assessed in aggregate and stratified by disease type, cancer stage, grade, tumour size, histology, and covariates.

Differences between those with and without weight loss will be assessed using the log-rank test.

#### Multivariate Cox regression

Cox regression will be used to estimate the adjusted hazard ratios (HR) for cancer or serious disease associated with weight loss recorded as a symptom.

The impact of choosing to restrict the follow-up period on the predictive value of weight loss will be explored by limiting the analysis by time period (0–6, 6–12, 12–18, and 18–24 months) and by including weight loss as a time dependent variable.

Age at index date, sex, ethnicity, IMD score, co-morbidity, smoking, and alcohol intake will be included, and the predictive value of other symptoms and investigations will be explored for (1) all cancers in aggregate, (2) cancer type, (3) by cancer stage, (4) by tumour size, (5) by grade of cancer and (6) serious disease type.

#### Performance of diagnostic strategies

To allow clinical guidance to be developed on how to rule-in or rule-out cancer or serious disease in adult patients (> 18 years) with unexpected weight loss, diagnostic accuracy measures will be calculated for investigative strategies including those described in the literature including the subgroups of (1) gender and (2) age-group.

## Plan for addressing confounding

### Aim 1: Descriptive study

Not required.

### Aim 2: Cohort analysis

Patients who have conditions which might explain the weight loss (e.g. co-morbidities at the time of entry to the cohort or planned dieting) will be included and the impact of their inclusion assessed in multivariate and sensitivity analyses.

Patients with coded weight loss will be matched with patients without a weight loss code based on GP practice to account for systematic biases in coding between practices.

Age at index date, sex, IMD score, co-morbidity, smoking, and alcohol intake will be adjusted for in the multivariate modelling.

## Plan for addressing missing data

### Aim 1: Descriptive study

Weight is cited as a missing variable in CPRD as GPs do not routinely measure weight in NHS primary care [8]. This descriptive analysis will add to our understanding of how often and when weight is recorded.

We will also describe the completeness of personal characteristics (as defined above) in relation to weight measurements and weight loss codes.

### Aim 2: Cohort analysis

As measurements appear to be too infrequent to allow us to identify weight loss from serial weight measurement data, the cohort design will make best use of the coded weight loss information available in CPRD. For this reason, we do not intend to impute missing weight measurement values in the primary analysis, although the feasibility of using multiple imputation to address missing covariate values will be explored [10].

## Discussion

Within this section, we expand on the protocol as submitted to ISAC to elucidate decisions made about study design and to report developments made since commencing the study. We have incorporated and expanded upon the “Limitations of the study design, data sources and analytical methods” section of the original ISAC protocol.

### Reliance on weight loss coding

It appears from our preliminary searches that weight measurement is infrequent for the majority of patients in primary care, most likely initiated by a concern for underlying disease or existing chronic disease management. This is consistent with studies that acknowledge weight measurement as a source of missing data in NHS primary care records [8]. Consequently, the detection of weight loss from serial weight measurements cannot be relied on as a method of defining weight loss. Our descriptive analysis is designed to identify whether a group of patients exists who undergo weight measurements more frequently, in which a future analysis involving serial weight measurements may be feasible. However, any subgroup is unlikely to be representative of the NHS primary care population. We have therefore chosen to focus on weight loss coding.

As with previous primary care studies using routinely collected data, an assumption will be made that the absence of a symptom code represents the absence of the symptom [5, 11]. This assumption has two major limitations: firstly, a coded entry is reliant on the patient visiting the GP and reporting the symptom; and secondly, that the GP chooses to enter the code in the record. Lack of the former would lead to an underestimation of the associated HR, and for the latter, selective recording of symptoms only deemed severe by the GP could lead to overestimated HRs. The latter is likely to differ by GP but cluster by GP practice, as GPs within the same practice are likely to have more similar approaches to coding. One method to address these limitations would be to analyse free-text entries to identify reported but uncoded symptoms, but at present CPRD does not allow requests for free-text entries and we will cite this as a weakness of our study [12]. We decided to adjust for age and sex in multivariate analysis as the association between weight loss and cancer is not established for these variables.

#### Sample size for cohort analysis

Progress since the initial ISAC application has established that there are 148,000 patients eligible patients aged > 18 years with an unexpected weight loss code as described in Appendix 1 (preliminary pilot work had suggested there was at least 30,000). This will therefore be the largest primary care CPRD cohort study using unexpected weight loss coding as the exposure variable. We originally calculated that only 2184 patients with weight loss are required to detect a hazard ratio of 2 at 99% power (0.05% alpha) using an enrolment ratio of 1:5. That is, a change in a cancer risk from a PPV of 1.5% in patients without weight loss to 3% in patients with weight loss. An alternative approach to estimating sample size is the number of Events Per Varaible in multivariate modelling. If 3% of patients with weight loss develop cancer the number of Events Per Variable will far exceed the minimum number of ten required for robust multivariate modelling. It is anticipated that the study will therefore have sufficient power for stratification by cancer type.

We aim to understand the association between weight loss and cancer in as much detail as the data permits. However, we accept it may not be possible to stratify for cancer stage or for other covariates with sufficient numbers remaining in each stratum. Cancer stage information is unsatisfactory in CPRD, which is why we have requested data linkage to the cancer registry (which will also be incomplete, but less so). Lifestyle covariates are non-essential for our main aim (to determine the predictive value of weight loss for cancer), and we will only perform analysis on sub-strata when numbers permit. Multiple imputation will be explored for these (and all other relevant missing) variables.

#### Investigation and referral outcomes

There remains uncertainty over the completeness of investigation and referral data until the descriptive analysis has been conducted. Data for laboratory investigations are likely to be more complete than data on radiological and endoscopic investigations, as laboratory investigations are commonly transmitted directly into the electronic health record from the laboratory whereas results for the other tests are not. Further linkage to the Diagnostic Imaging Dataset (for radiology activity) and Hospital Event Statistics (for endoscopy activity) may be necessary if these data are judged to be incomplete following the descriptive analysis, which would allow a formal comparison of data completeness to be conducted between these datasets and CPRD.

## Implications

A second cohort study using American primary care data is also in set-up to assess whether there is greater value in defining weight loss using serial weight measurements rather than a reliance on patient reported weight loss and a GP entered code. In particular, this study aims to establish whether weight loss detected using change in serial weight measurements leads to less advanced disease at diagnosis.

Together, these studies will provide the largest reported retrospective cohorts of primary care patients with unexpected weight loss used to understand the association between unexpected weight loss and serious disease including cancer. We hope our findings will directly inform international guidelines for the management of unexpected weight loss in primary care populations.

## Abbreviations

BMI: Body mass index
CPRD: Clinical Practice Research Datalink
IMD: Index of Multiple Deprivation
ISAC: Independent Scientific Advisory Group (to the CPRD)
Medcode: The CPRD unique code for the medical term selected by the GP
NCDR: National Cancer Data Repository
NICE: National Institute for Health and Care Excellence
Read code: The standard clinical terminology system used in general practice in the UK
